# The association of circulating irisin with metabolic risk factors in Chinese adults: a cross-sectional community-based study

**DOI:** 10.1186/s12902-019-0479-8

**Published:** 2019-12-27

**Authors:** Lizhi Tang, Yuzhen Tong, Fang Zhang, Guilin Chen, Yun Cong Zhang, John Jobin, Nanwei Tong

**Affiliations:** 1 Division of Endocrinology and Metabolism, West China Hospital of Sichuan University, No. 37 Guoxuexiang, Chengdu, 610041 Sichuan China; 2 Brescia University College, Western University, London, Ontario Canada; 3 West China School of Medicine, West China Hospital of Sichuan University, Chengdu, Sichuan China; 4 College of medicine, University of Saskatchewan, Saskatoon, Saskatchewan Canada

**Keywords:** Irisin, Obesity, Insulin resistance, Myokines

## Abstract

**Background:**

Irisin is a myokine that leads to increased energy expenditure by stimulating the browning of white adipose tissue. We aimed to investigate the association of serum irisin levels with metabolic parameters in middle aged Chinese population.

**Methods:**

The study was based on a cross-sectional analysis of data from 524 nondiabetic subjects aged 40~65. All participants were recruited from a screening survey for Metabolic Syndrome in a community in Southwest China, including 294 subjects categorized as overweight (defined as BMI≧25 kg/m^2^) and 230 subjects as normal control (defined as 18.5≦BMI < 25 kg/m^2^). Serum irisin concentration was quantified by enzyme linked immunosorbent assay (ELISA). The relationship of irisin with metabolic factors was determined by Pearson correlation. Multivariate linear regression was used to analyze the association of irisin with insulin resistance. Logistic regression was performed to assess the association of irisin with odds of overweight.

**Results:**

Serum irisin levels were significantly lower in nondiabetic overweight subjects compared with control (11.46 ± 4.11vs14.78 ± 7.03 μg/mL, *p* = 0.02). Circulating irisin was positively correlated with quantitative insulin sensitivity check index (QUICKI, *r* = 0.178, *p* = 0.045) and triglycerides *(r* = 0.149, *p* = 0.022); while irisin was negatively correlated with waist circumference (WC, *r* = − 0.185, *p* = 0.037), waist-to-hip ratio (WHR, *r* = − 0.176, *p* = 0.047), fasting insulin (*r =* − 0.2, *p* = 0.024), serum creatinine (*r* = − 0.243, *p* = 0.006), homeostasis model assessment for insulin resistance (HOMA-IR, *r* = − 0.189, *p* = 0.033). Multiple linear regression showed that irisin was inversely associated with HOMA-IR (β = − 0.342 ± 0.154, *p* = 0.029). Higher irisin was associated with decreased odds of being overweight (OR = 0.281, β = − 1.271, *p* = 0.024).

**Conclusions:**

We found that serum irisin levels were lower in overweight subjects. Moreover, serum irisin levels were inversely correlated with adverse metabolic parameters including WC, WHR, creatinine, HOMA-IR and fasting insulin, suggesting that irisin may play a role in obesity related insulin resistance.

## Background

Obesity is dramatically on the rise and has become an epidemic globally. The prevalence of obesity-related morbidities also increased invariably as a result of it, including type 2 diabetes mellitus (T2DM), metabolic syndrome (MS), hypertension, chronic kidney disease (CKD), cardiovascular disease (CVD), heart failure and cancer [1]. Sedentary lifestyle and high calorie intake result in excess of energy. Obesity is necessarily the consequence of a long-term imbalance between energy intake and expenditure, which result in insulin resistance and an imbalance in glucose metabolism that lead to the development of T2DM [2, 3].

During the past decade, skeletal muscle has been identified as a secretory organ [4]. Myokines include cytokines and other peptides produced, expressed and released by skeletal muscle that creates endocrine effects, and these myokines are responsible for the immediate and chronic benefits of exercise on metabolic diseases [1, 4]. Irisin is a recently discovered myokine, it is a cleaved membrane protein encoded by the fibronectin type III domain containing 5 (FNDC5) gene [5]. Recent studies showed irisin can stimulate the expression uncoupling protein-1 (UCP-1), result in browning of white adipose tissue, thereby stimulating energy expenditure [6]. Expression of peroxisome proliferator-activated receptor gamma coactivator-1α (PGC-1α) of skeletal muscle can be induced by exercise which can stimulate the irisin secretion by endorsing the expression of FNDC5 [7, 8]. As a result, irisin is emerging as a promising therapeutic target for the treatment of obesity associated metabolic diseases [6].

Correlations between circulating irisin levels and adverse metabolic phenotypes have been analyzed in humans by several studies with conflicting results [9–13]. Positive association between circulating irisin levels at baseline or FNDC5 mRNA expression and body mass index (BMI) was demonstrated in most of the studies [9, 11]. However, another study found a negative association of circulating irisin with BMI, waist-to-hip ratio (WHR), and fat mass; decreased irisin concentration and FNDC5 gene expression were found in adipose tissue and muscle from patients with obesity [12] . Moreno-Navarrete JM and coworkers reported circulating irisin levels were negatively associated with obesity and insulin resistance in men [14]. A recent study found that irisin is inversely associated with fasting insulin level in obese Chinese adults but was not associated with BMI and waist circumference (WC) [13].

Therefore, the aims of our study are to evaluate the circulating serum irisin levels in overweight and control subjects and also to elucidate possible relationships of serum irisin levels with anthropometric and metabolic parameters in a population of Chinese adults.

## Methods

### Study participants

All participants were recruited from a screening survey for Metabolic Syndrome in Yinchao community of Chengdu, Sichuan province of China, between September and November 2011. Information about lifestyle and medications were obtained by questionnaire [15]; anthropometrical measurements were measured by trained physicians. 75-g oral glucose tolerance test (OGTT) was performed; subjects with T2DM and newly diagnosed T2DM were excluded. We recruited 524 nondiabetic subjects with full survey and biochemical data including 230 subjects with normal BMI (controls, defined as 18.5 ≦ BMI < 25 kg/m^2^) and 294 subjects overweight (defined as BMI ≧ 25 kg/m^2^) according to WHO definition of overweight [16].

### Anthropometric measurements

Individual height, weight, waist circumferences (WC) were measured by trained physicians; BMI was calculated as weight in kilograms divided by height in squared meters (kg/m^2^); WC was measured in the standing position, midway between the iliac crest and the lower costal margin and hip circumference (HC) was the widest diameter around the most prominent points of hip pelvis. Waist-to-hip ratio (WHR) was calculated by dividing WC (cm) by HC (cm). Blood pressure was measured three times by a mercury sphygmomanometer at 5-min intervals after 10-min rest in seated position and the mean value was used.

### Biochemical measurements

Blood samples were collected after a 12-h overnight fasting, instantly centrifuged; serum and plasma were used for biochemical marker analysis. Serum triglycerides (TG), total cholesterol (TC), high-density lipoprotein (HDL-C) cholesterol and low-density lipoprotein (LDL-C) cholesterol and serum creatinine (Scr) were measured by enzymatic methods with commercial reagent sets (Boehringer Mannheim). Blood glucose including fasting plasma glucose (FPG), 30-min plasma glucose (30-minPG) and 2-h glucose (2-hPG) after OGTT were measured by a glucose oxidase procedure. Glycosylated hemoglobin A1c (HbA1c, %) was measured by high performance liquid chromatography (HPLC, Bio-Rad D-10 hemoglobin A1C radiometer) [15].Serum fasting insulin (Fins), 30-min insulin (30-minIns) and 2-h insulin (2-hIns) after OGTT were measured by ELISA kits (cobas e411, Toche Company, Switzerland). Serum irisin levels were measured by using the enzyme-linked immunosorbent assay (ELISA) kits (BioVision, Milpitas, CA), according to the manufacturer’s instructions. The sensitivity of the assay was 1 ng/mL. The coefficients of variation for intra- and inter-assay were 8 and 10% respectively [1].

Insulin resistance was estimated by the homeostasis model assessment for insulin resistance (HOMA-IR) which was calculated as fasting insulin (mU/L) x fasting glucose (mmol/L)/22.5 [17]. The quantitative insulin sensitivity check index (QUICKI) was calculated as 1/[log fasting insulin (mU/mL) + log fasting glucose (mg/dL)] [18].

### Statistical analysis

Shapiro-Wilk test was used for checking normality of all variables. Continuous variables were expressed as mean ± SD. Student’s *t* test was used for normally distributed variables. Variables which did not fulfill the normality assumptions were log-transformed before parametric analysis. Correlation between irisin and metabolic parameters was assessed by age and sex adjusted Pearson correlation analysis. Multiple linear regression was used to assess the association of irisin with HOMA-IR. Logistic regression was applied to determine the association of irisin with odds of overweight (coded as 1), we coded controls = 0 (defined as 18.5 ≦ BMI < 25 kg/m^2^), overweight = 1 (defined as BMI ≧ 25 kg/m^2^). Odds ratios (ORs) and 95% confidence intervals (CIs) were calculated for odds of overweight with relation to irisin. Sex, age smoking, alcohol and medication use were adjusted for regression model. Statistical analysis was performed by SAS 9.2 software (SAS Institute Inc., Cary, NC27513, USA). All tests were two-sided with *p* < 0.05 considered as statistically significant.

## Results

### Characteristics of the study population

The clinical characteristics and biochemical data of the controls and subjects with overweight are summarized in Table 1**.**

**Table 1:** Characteristics of the study population

	Controls (*n* = 230)	Overweight (*n* = 294)	*p*
Age (years)	56.74 ± 8.06	57.14 ± 8.86	0.08
Height (cm)	160.19 ± 7.64	157.61 ± 7.85	0.154
Weight (kg)	59.21 ± 9.09	65.58 ± 9.74	< 0.0001
BMI (kg/m^2^)	23 ± 2.49	26.37 ± 2.85	< 0.0001
WC (cm)	77.16 ± 6.72	87.42 ± 6.35	< 0.0001
WHR	0.84 ± 0.06	0.89 ± 0.05	< 0.0001
SBP (mmHg)	115.8 ± 16.04	130.89 ± 17.58	< 0.0001
DBP (mmHg)	72.68 ± 9.54	76.59 ± 11.53	< 0.0001
TG (mmol/L)	1.48 ± 1.21	1.95 ± 1.41	< 0.0001
TC (mmol/L)	4.59 ± 0.86	5.06 ± 0.98	< 0.0001
HDL-C (mmol/L)	1.59 ± 0.39	1.42 ± 0.3	< 0.0001
LDL-C (mmol/L)	2.82 ± 0.69	2.83 ± 0.66	0.0038
FPG (mmol/L)	5.16 ± 1.05	5.58 ± 1.26	0.0004
30-min PG (mmol/L)	9.19 ± 2.38	10.47 ± 2.89	< 0.0001
2-h PG (mmol/L)	7.01 ± 2.93	9 ± 4.23	< 0.0001
HbA1c (%)	5.54 ± 0.55	5.59 ± 0.73	0.02
Fins (mIU/mL)	6.25 ± 3.96	9.65 ± 5.49	< 0.0001
30-min Ins (mIU/mL)	51.06 ± 34.42	65.94 ± 44.49	< 0.0001
2-h Ins (mIU/mL)	37.32 ± 27.26	56.36 ± 36.42	< 0.0001
HOMA-IR	1.45 ± 1	2.45 ± 1.86	< 0.0001
QUICKI	0.31 ± 0.05	0.27 ± 0.04	< 0.0001
Scr (umol/L)	61.61 ± 16.02	69.43 ± 17.22	< 0.0001
Irisin (ug/mL)	14.78 ± 7.03	11.46 ± 4.11	0.02

*BMI* body mass index, *WC* waist circumference, *WHR* waist to hip ratio, *TG* triglycerides, *TC* total cholesterol, *SBP* systolic blood pressure, *DBP* diastolic blood pressure, *HDL-C* high density lipoprotein cholesterol, *LDL-C* low density lipoprotein cholesterol, *FPG* fasting plasma glucose, *PG* plasma glucose, *Fins* fasting insulin, *Ins* insulin, *HOMA-IR* homeostatic model for assessment of insulin resistance, *QUICKI* quantitative insulin-sensitivity check index, *Scr* serum creatinine

Compared to controls, subjects with overweight had significantly higher BW, BMI, WC, WHR, systolic blood pressure (SBP), diastolic blood pressure (DBP), TC, triglycerides, LDL-C, FPG, 30-minPG, 2-hPG, Fins, 30-minIns, 2-hIns, HbA1c, HOMA-IR, serum creatinine (Scr), lower HDL-C and QUICKI. Serum irisin levels were significantly lower in nondiabetic overweight subjects compared to control (11.46 ± 4.11 v s14.78 ± 7.03 μg/mL, *p* = 0.02).

### Correlation of irisin with anthropometric and biochemical parameters

Pearson’s correlation analysis for serum irisin with metabolic parameters are shown in Table 2. Circulating irisin level was positively correlated with QUICKI (*r* = 0.178, *p* = 0.045) and triglycerides (*r* = 0.149, *p* = 0.022), but was negatively correlated with WC (*r* = − 0.185, *p* = 0.037), WHR (*r* = − 0.176, *p* = 0.047), Fins (*r* = − 0.2, *p* = 0.024), Scr (*r* = − 0.243, *p* = 0.006) and HOMA-IR (*r* = − 0.189, *p* = 0.033). Irisin was not correlated with cholesterol (TC, HDL-C and LDL-C), glucose (fasting glucose, postprandial glucose and HbA1C) and blood pressure (SBP and DBP).

**Table 2:** Correlation of irisin with anthropometric and metabolic parameters

	*r*	*p*
Weight	− 0.09	0.313
BMI	−0.124	0.165
WC	−0.185*	0.037
WHR	−0.176*	0.047
SBP	−0.087	0.329
DBP	0.02	0.826
TG	0.149*	0.022
TC	−0.026	0.775
HDL-C	0.046	0.608
LDL-C	0.03	0.736
FPG	0.023	0.799
30-min PG	−0.11	0.215
2-h PG	−0.115	0.196
HbA1c	0.038	0.671
Fins	−0.2*	0.024
30-min Ins	0.062	0.49
2-h Ins	−0.108	0.225
HOMA-IR	−0.189*	0.033
QUICKI	0.178*	0.045
Scr	−0.243*	0.006

*BMI* body mass index, *WC* waist circumference, *WHR* waist to hip ratio, *TG* triglycerides, *TC* total cholesterol, *SBP* systolic blood pressure, *DBP* diastolic blood pressure, *HDL-C* high density lipoprotein cholesterol, *LDL-C* low density lipoprotein cholesterol, *FPG* fasting plasma glucose, *PG* plasma glucose, *Fins* fasting insulin, *Ins* insulin, *HOMA-IR* homeostatic model for assessment of insulin resistance, *QUICKI* quantitative insulin-sensitivity check index, *Scr* serum creatinine. r, Pearson correlation coefficient. *p < 0.05 is significant.

### Multiple linear regression of irisin with HOMA-IR

Multiple linear regression was performed to assess the association of irisin with HOMA-IR (Table 3). Our results showed that irisin was negative associated with HOMA-IR (β = − 0.342, *p* = 0.029) after adjustment of age, sex, smoking, alcohol and medication use.

**Table 3:** Multiple linear regression of irisin with HOMA-IR

	β ± SE of β	*t*-Value	*p*
Age	0.014 ± 0.006	2.6	0.01
Sex	0.153 ± 0.113	1.35	0.181
BMI	0.063 ± 0.016	3.93	0.0002
Irisin	−0.342 ± 0.154	−2.22	0.029

Sex, age smoking, alcohol and medication use were adjusted for model. *BMI* body mass index, β regression coefficient, *SE* standard error; *p* < 0.05 is significant.

### Multivariate logistic regression analysis of irisin with odds of overweight

In order to analyze the association of irisin with odds of overweight, logistic regression was performed. We coded overweight = 1 (BMI≧25 kg/m^2^) and normal controls = 0 (18.5≦BMI < 25 kg/m^2^) as shown in Table 4. Higher level of irisin was significantly associated with decreased odds of overweight with odds ratio of 0.281 (β = − 1.271, 95% CI: 0.093 ~ 0.851, *p* = 0.024).

**Table 4:** Logistic regression analysis of irisin with odds of overweight

	β	OR	95% CI	*p*
Model: Overweight (BMI≧25 kg/m^2^) = 1, Controls = 0				
Age	0.035	1.035	(0.99~1.083)	0.132
Sex	−1.421	0.242	(0.106~0.548)	0.000
Irisin	−1.271	0.281	(0.093~0.851)	0.024

Age, sex, smoking, alcohol and medication use were adjusted. β regression coefficient, *OR* odds ratio; 95% CI, 95% confidence interval, *p* < 0.05 is significant.

## Discussion

In the present study, we found that serum irisin levels were significantly lower in overweight subjects compared to controls in middle aged Chinese. Circulating irisin concentration was negatively correlated with adverse metabolic parameters including WC, WHR, fasting insulin, HOMA-IR and serum creatinine. Moreover, multiple linear regression revealed that irisin was significant in an inverse relationship with HOMA-IR. In addition, logistic regression showed that higher irisin was associated with decreased odds of overweight.

In accordance with our finding, Liu et al. also reported decreased irisin level in obese Han Chinese [19]. Several studies have implicated the role of PGC-1α in pathogenesis of obesity and T2DM [20, 21]. Moreover, PGC-1α expression and its activity were significantly down-regulated in skeletal muscles in patients with obesity and T2DM [21, 22]. Irisin was discovered as a PGC-1α activated messenger of myocytes that linked physical inactivity, obesity and diabetes [20]. Thus, it is possible that lower levels of irisin in overweight observed in our study might be caused by impaired PGC-1α expression and functions in their muscle tissues.

In study conducted in patients with insulin resistance, irisin levels were determined to increase with the insulin resistance and decrease as insulin sensitivity increases [23]. On the contrary, we found a significant negative correlation of irisin with fasting insulin and HOMA-IR, positive correlation with insulin sensitivity index QUICKI. In agreement with our findings, Yan and coworkers showed that irisin was negatively associated with fasting insulin in a large Chinese population with MS [24]. Another study by Shi et al. showed that elevated circulating irisin was associated with lower risk of insulin resistance indirectly through lowering fasting insulin in obesity [13]. The negative association of irisin with HOMA-IR and insulin in patients with nonalcoholic fatty liver disease (NAFLD) was also demonstrated by Shanaki M et al [25] A negative association of HOMA-IR and circulating irisin levels in young girls demonstrated in a recent study indicate that the onset of obesity, insulin resistance and T2DM might be delayed by the irisin secretion at an early age [26]. In our study, the negative correlation of irisin with markers of insulin resistance indicated that decreased irisin expression in response to decreasing insulin sensitivity and disturbance in metabolisms associated with obesity.

On the contrary, irisin has been positively associated with HOMA-IR [27]. Researchers found that irisin were positively associated with fasting insulin and blood glucose in healthy individuals, and in women with polycystic ovary syndrome and in children with obesity but without T2DM [10, 12, 27]. A meta-analysis revealed that irisin concentration was positively associated with insulin resistance in adults who do not have T2DM [28]. The negative association between irisin and HOMA-IR observed in our study could be secondary results of impaired PGC-1α function in obesity. As we know, PGC-1α can stimulate the expression of irisin, which is induced by exercise and exerts profound activity in the WAT, stimulating browning of WAT and UCP1 expression. Importantly, this causes a significant increase in total body energy expenditure and resistance to obesity-linked insulin resistance [7].

Different types of diabetes may have different levels of irisin. The levels of irisin in type 1 diabetes are not fully defined yet, one study showed the level of irisin in patients with type 1 diabetes was higher than control [29], while another study showed opposite results [30]. Most studies have shown that irisin levels were lower in patients with T2DM [31, 32]. A meta-analysis of 1289 patients with T2DM and 834 controls showed lower irisin in patients with T2DM [32]. It is possible to conclude that irisin hormone is not only associated with exercise but also with hormones, insulin resistance, inflammation and autoimmunity.

Current study on irisin concentrations and adiposity parameters remains controversial [9–12, 33]. In accordance with our findings, some studies found a significant negative correlation of circulating irisin with WC [24] and WHR [12] in nondiabetic individuals. However, contradictory to our results most studies have revealed a positive correlation of serum irisin levels with BMI [9, 11, 31] and WC [10] in nondiabetic individuals. Van Marken Lichtenbelt et al. showed that the amount of BAT was significantly decreased in association with obesity, with a negative linear relationship between BAT, BMI and percent body fat [34]. Although we found there was no association between irisin and BMI; Pearson correlation showed that irisin was inversely associated with WC and WHR; suggesting abdominal obesity could be a link between decreased irisin and insulin resistance.

A population-based cohort included 967 non-diabetic people living in Germany, found irisin had a significant association with favorable lipid profile; especially irisin was negatively associated with total cholesterol concentration [35]. Buscemi S, et al. also found a positive association between HDL-cholesterol concentrations and irisin concentration [36]. However, we found a positive association between irisin and triglycerides, no association with total cholesterol or HDL-C.

We also found that irisin was negatively correlated with serum creatinine. Accordingly, Ates I et al. showed irisin was negatively associated with serum creatinine among patients with type 1 diabetes [29]. A previous study conducted in patients with chronic renal failure, irisin levels were determined to be negatively correlated with creatinine, which is considered to result from the inhibition of FNDC5 by indoxyl sulphate which is a uremic toxin [37]. The negative association between irisin and creatine suggest that creatinine may have a role in the pathophysiology of irisin or vice versa. Further studies are needed to uncover the possible underlying mechanism for this.

We do have some limitations in this study. Firstly, the cross-sectional study was unable to give information on prospective changes for irisin and its association with each metabolic parameter. Secondly, our sample size was relatively small, therefore we did not further sub-divide overweight patients by obesity level. So the conclusion should only be generalized to overweight rather than different grade of obesity. Moreover, there has been controversies regarding available irisin ELISA kits, including antibody specificity of antibody, its cross-reactivity with FNDC5 and the wide range of irisin levels between different studies [36, 38]. In addition, different ethnicity and lifestyle may have different results; generalization beyond Asian populations should be interpreted with caution.

## Conclusions

We found that serum irisin levels were decreased in overweight, which was presumably related to impaired muscle PGC-1α expression or dysfunction among these subjects. Moreover, serum irisin level was negatively associated to HOMA-IR and fasting insulin, suggesting that irisin may play a role in obesity related insulin resistance. Thus, modification of circulating irisin level may help in the management of obesity and related metabolic diseases. However, in order to elucidate the potential of irisin as a valuable drug target in human disease conditions, additional studies on the aspects of secretion and metabolism of irisin are required.

## Data Availability

The datasets used and/or analysed during the current study are available from the corresponding author on reasonable request.

## Abbreviations

CKD: Chronic kidney disease
CVD: Cardiovascular disease
FNDC5: Fibronectin type III domain containing 5
FPG: Fasting plasma glucose
HC: Hip circumference
HDL-C: High-density lipoprotein cholesterol
HOMA-IR: Homeostasis model assessment for insulin resistance
LDL-C: Low-density lipoprotein cholesterol
MS: Metabolic syndrome
OGTT: Oral glucose tolerance test
PG: Plasma glucose
PGC-1α: Peroxisome proliferator-activated receptor gamma coactivator-1α
QUICKI: Quantitative insulin sensitivity check index
Scr: Serum creatinine
T2DM: Type 2 diabetes mellitus
TC: Total cholesterol
TG: Triglycerides
WC: Waist circumference
WHR: Waist-to-hip ratio
